# Circumventing Inner Filter Effects in Ultrafast Near-Infrared II (NIR-II) Up-Conversion Spectroscopy Using Front-Face Optical Configuration

**DOI:** 10.1021/acs.analchem.6c05839

**Published:** 2026-09-21

**Authors:** Tse-Hua Hsu, Hao-Chun Huang, Yen-Chang Chen, Yang-Hsiang Chan, Li-Kang Chu, I-Chia Chen, Bo-Han Wu

**Affiliations:** † Department of Chemistry, 34881 National Tsing Hua University, 101, Sec. 2, Kuang-Fu Road, Hsinchu 300044, Taiwan; ‡ Department of Applied Chemistry, 34914 National Yang Ming Chiao Tung University, 1001, University Road, Hsinchu 300093, Taiwan

## Abstract

Self-absorption is fatal in up-conversion ultrafast spectroscopy, severely suppressing the fluorescent signal. To conquer this problem, we implemented a front-face optical configuration in fluorescence up-conversion spectroscopy to accurately record the intrinsic excited-state dynamics of near-infrared II (NIR-II) fluorophores, significantly minimizing the optical path length of sample absorption. Concurrently, the inner filter effect (IFE) was greatly prohibited; i.e., the intrinsic fluorescence evolution can be correctly recorded without self-absorption. By combining this optical geometry with the high temporal resolution of the up-conversion technique, we successfully recorded the ultrafast dynamics of the benchmark organic dye IR1061 and the porphyrinoid S2F-2H^+^ at 1235 nm, in terms of their decay lifetimes of 31 ± 1 ps and 74 ± 3 ps, respectively. Notably, despite a nearly zero Stokes shift (64 cm^–1^) and strong spectral overlap of the absorption and emission contours of S2F-2H^+^, the up-conversion detection using front-face geometry leads to a superb signal-to-noise ratio of *ca*. 334. This optical configuration provides a promising tool for probing NIR-II fluorescence systems, especially for investigating highly self-absorbing materials and paving the way for the rational design of high-performance NIR-II fluorophores.

## Graphical abstract

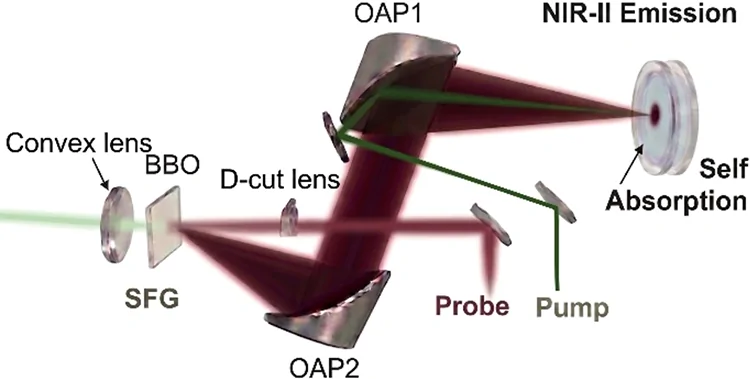

## Introduction

The development of organic fluorophores emitting in the second near-infrared window (NIR-II, 900–1700 nm) has emerged as a pivotal frontier in biomedical imaging.^1,2^ Compared to traditional imaging windows in the visible (400–700 nm) and first near-infrared (NIR-I, 700–900 nm) regions, NIR-II emission offers superior tissue penetration depth and significantly reduced photon scattering,^3^ effectively minimizing the tissue autofluorescence and achieving an exceptional signal-to-noise ratio for high-resolution deep-tissue visualization. These advanced molecular probes enable a wide range of applications, including photothermal therapy,^4^ real-time intraoperative guidance,^5^ precise tumor identification,^6^ and so on.

High-performance organic NIR-II fluorophores face severe physical constraints.^7,8^ According to the energy gap law, the excited-state energy is more easily dissipated through nonradiative decays, such as molecular vibrations, when the energy gap narrows.^9^ Consequently, the quantum yield of traditional long-wavelength dyes is typically extremely low.^10,11^ Recent research has focused on enhancing structural rigidity to minimize reorganization energy.^12^ While this vertical energy level strategy effectively suppresses vibrational interference and boosts fluorescence efficiency, it inevitably leads to a minimal Stokes shift.^13,14^ This high spectral overlap of absorption and emission contours causes a severe self-absorption effect, where the emitted photons are strongly reabsorbed by neighboring ground-state molecules before being detected. This self-absorption, i.e., the inner filter effect (IFE), is a critical issue in ultrafast fluorescence spectroscopy and hampers the acquisition of the intrinsic excited-state dynamics. Therefore, developing innovative ultrafast spectroscopic techniques to overcome self-absorption-induced loss of fluorescence intensity and to precisely resolve the excited-state evolution of NIR-II molecules has become the core technical challenge of these studies.

To address these critical challenges, front-face and reflection collection geometries have been recognized as effective strategies for suppressing self-absorption and inner filter effects in time-resolved photoluminescence spectroscopy.^15–17^ While previous ultrafast studies applied front-face optics to UV–visible Kerr gating^17^ or transient grating spectroscopy,^16^ adapting this geometry to a NIR-II fluorescence up-conversion system is far more challenging. This difficulty mainly stems from the strict optical alignment required for NIR-II beams and their spatial overlap in nonlinear gating crystals.

Unlike traditional transmission geometries,^18^ the front-face configuration for fluorescence up-conversion spectroscopy that we implemented significantly minimized the optical path length of the sample absorption and the inner filter effect. By combining this optical geometry with the high temporal resolution of the up-conversion technique, we successfully overcame the signal masking caused by spectral overlap and pursued the precise characterization of the radiative decay and excited-state dynamics of high-performance NIR-II molecules. This setup was utilized to investigate the ultrafast excited-state dynamics of two key NIR-II molecular systems, the benchmark organic dye **IR1061** and the porphyrinoid **S2F-2H** ^**+**^,^14,19^ which have the molecular structures shown in Figure 1a,b, respectively. Both molecules display broad optical absorption spanning from the visible to the NIR-II region, as evidenced by their steady-state absorption spectra in Figure 1c–f. Notably, both molecules exhibit absorption and emission profiles within the NIR-II window with minimal Stokes shifts, as shown in Figure 1e,f, respectively, and they are especially prominent in S2F-2H^+^. Our primary objective is to elucidate their ultrafast radiative deactivation pathways from the first excited state (S_1_) to the ground state. Consequently, this front-face optical configuration (Figure 2) provides a promising tool for the accurate characterization of diverse NIR-II luminescent molecules, even those possessing intense self-absorption.

**Figure 1:**
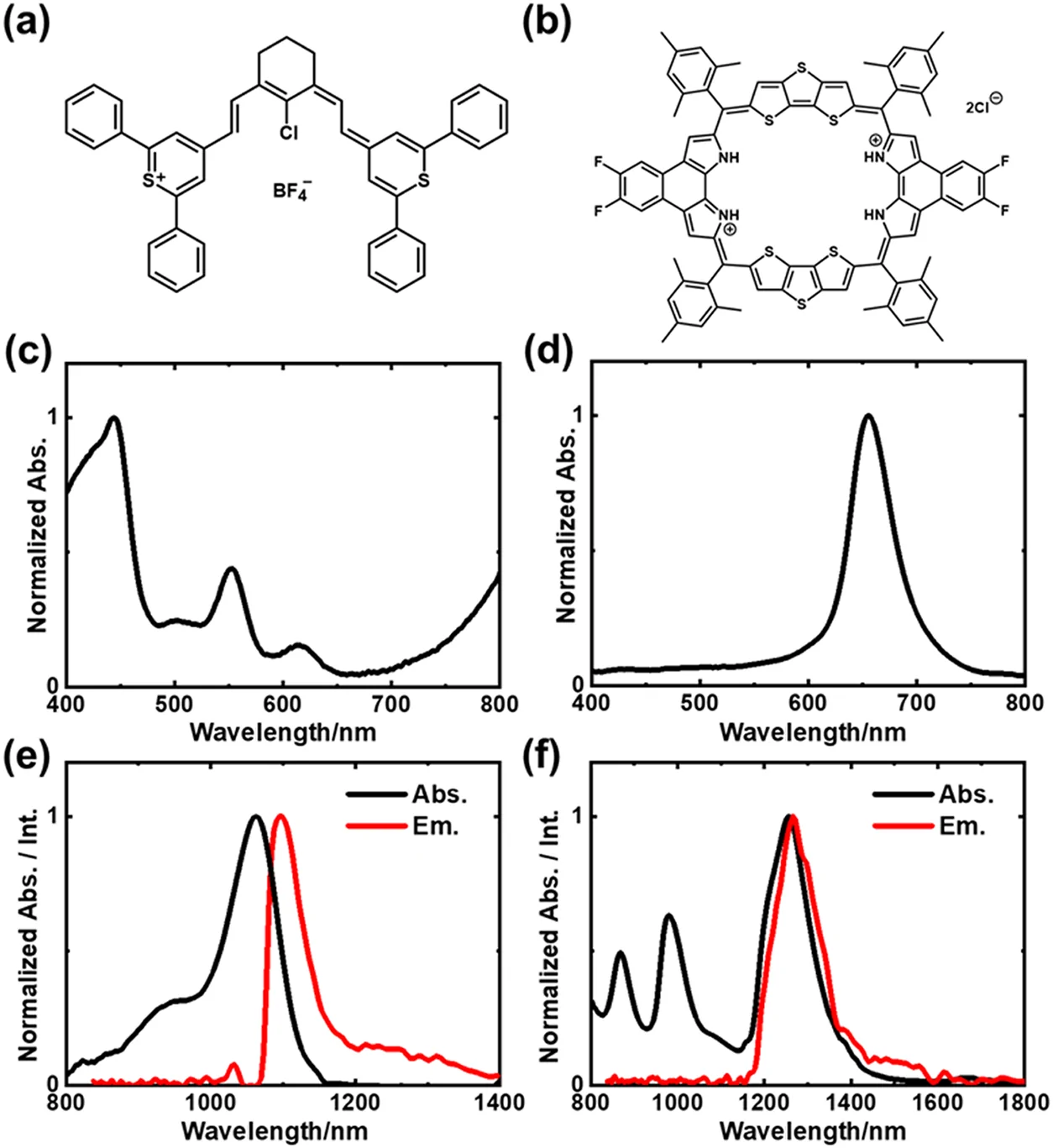
Molecular structures of (a) IR1061 and (b) S2F-2H^+^. Normalized visible absorption spectra of (c) IR1061 and (d) S2F-2H^+^. (e) Normalized NIR absorption (black) and emission (red) spectra of IR1061. (f) Normalized NIR absorption (black) and emission (red) spectra of S2F-2H^+^. The visible absorption spectra were recorded by a visible spectrometer. The NIR absorption spectra were recorded in the FTIR transmission mode, while the static emission spectra were obtained by integrating ssFTIR signals (0–1 μs) upon 1064 nm excitation.

**Figure 2:**
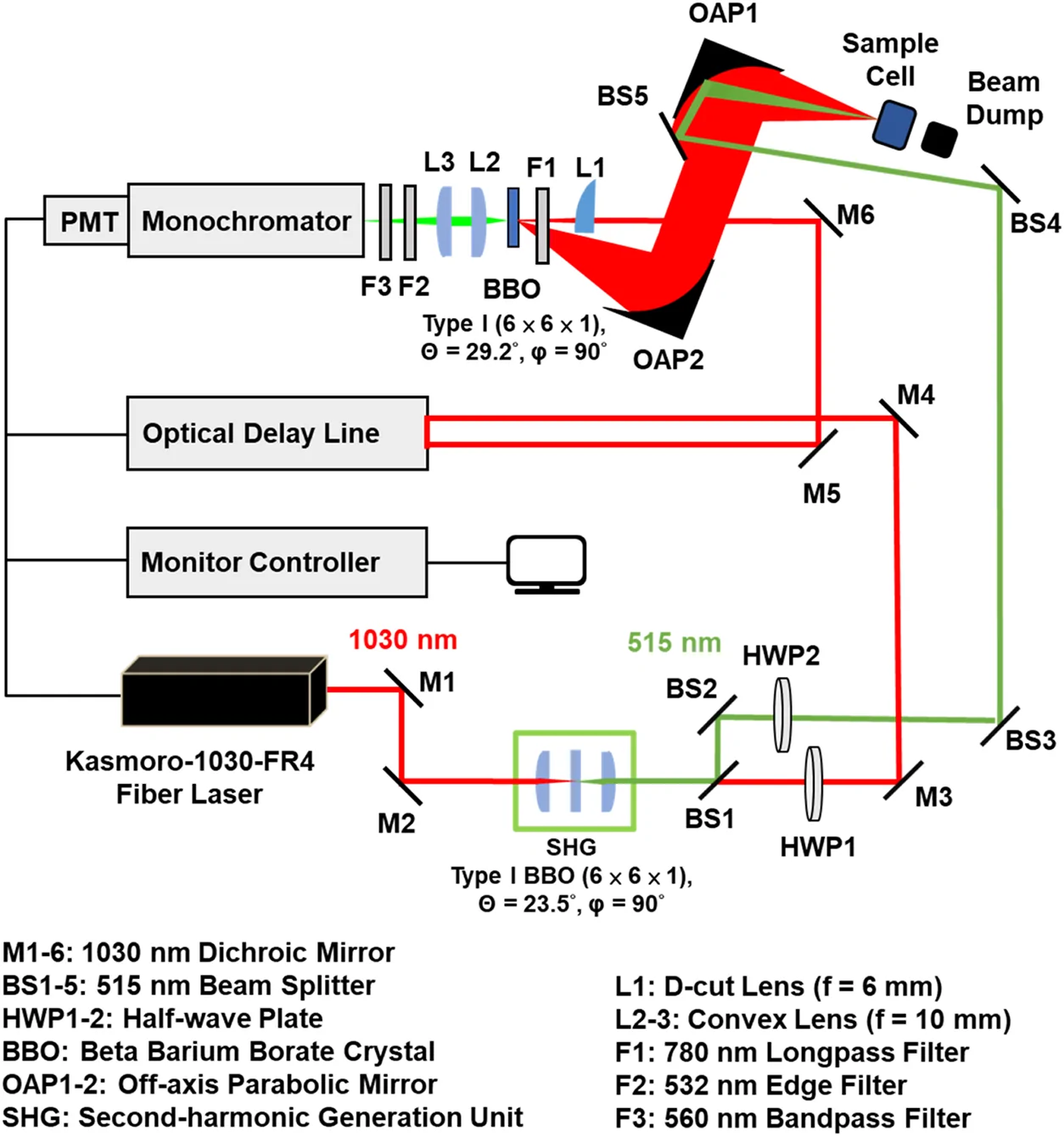
Schematic diagram of the experimental setup for the ultrafast NIR-II up-conversion spectroscopy via front-face geometry.

## Experimental Section

### Materials

4-[2-[2-Chloro-3-[(2,6-diphenyl-4*H*-thiopyran-4-ylidene)­ethylidene]-1-cyclohexen-1-yl]­ethenyl]-2,6-diphenylthiopyrylium tetrafluoroborate (IR1061, 80%, Sigma-Aldrich) and dichloromethane (DCM, 99.8%, Merck KGaA) were used without further purification. The S2F-2H^+^ molecule was prepared and purified as described previously.^14^ Stock solutions of IR1061 (140 μM) and S2F-2H^+^ (280 μM) were prepared by dissolving 1.04 mg and 4.11 mg of the powders, respectively, in 10.0 mL of DCM and sonication for 10 min.

### Steady-State Visible Absorption Spectroscopy

Visible absorption spectra were measured with a homemade absorption spectrometer as previously reported.^20^ A tungsten-halogen lamp (QTH10/M, Thorlabs, 400–2200 nm) as a probe beam was introduced by an optical fiber (BF19Y2HS02, Thorlabs, 250–1200 nm) to the sample compartment with an optical collimator (74-UV, Ocean Optics, 180–2500 nm). After passing through the sample cuvette, the light was delivered to the spectrometer (USB4000-UV–vis, Ocean Optics) via another optical collimator (74-UV, Ocean Optics, 180–2500 nm) coupled with an optical fiber (QP400–025-SR, Ocean Optics, 200–1100 nm). The liquid samples of IR1061 and S2F-2H^+^ dissolved in DCM were loaded in a 1 cm path length quartz cuvette for steady-state visible absorption acquisition, as shown in Figure 1c,d, respectively.

### Steady-State NIR Absorption Spectroscopy

The steady-state NIR absorption spectra were acquired in the transmission mode using Fourier-transform near-infrared spectroscopy (FTNIR, Vertex 80, Bruker). Briefly, an internal tungsten lamp served as the NIR light source, and the interferometer was equipped with a CaF_2_ beam splitter. The liquid samples of IR1061 and S2F-2H^+^ dissolved in DCM were loaded into a 2 mm path length quartz cuvette (104002F-10–40, Hellma Analytics) for absorption characterization. The spectra were acquired in the range of 5000–12,000 cm^–1^ with a spectral resolution of 64 cm^–1^ upon 16 scans for a better signal-to-noise ratio.

### NIR Emission Spectroscopy

The NIR emission spectra were measured by step-scan Fourier-transform infrared spectroscopy (ssFTIR, Vertex 80, Bruker) according to previous reports.^21–25^ The sample was loaded into a 1 cm path length quartz cuvette and mounted at the focal point of the parabolic mirror on the side of the interferometer for collecting the external emission. A fundamental 1064 nm Nd:YAG laser (DPS-1064-Q-24mJ, CNI Laser), operating at a repetition rate of 200 Hz, was used to excite the NIR absorption bands of IR1061 and S2F-2H^+^. An InGaAs-biased detector (DET10C2, Thorlabs) collected the scattering of the 1064 nm laser to serve as a trigger. A liquid nitrogen-cooled HgCdTe detector was employed to collect the infrared emission evolution and to transduce the photon signal into voltage. This signal at all optical retardation points was ac-coupled and further amplified by a factor of 10 and electronically filtered at a range of 100 Hz–1 MHz using an external voltage amplifier (SR560, Stanford Research Systems). Then the signal was processed by an analog-to-digital convertor (20 MHz, 14 bits, Bruker). A 1064 nm notch filter (NF03–532/1064E-25, Semrock) and a high-pass filter (RG-830, SCHOTT) were positioned in the optical path to prevent laser scattering and stray light from reaching the HgCdTe detector. The temporal profile at each optical retardation point was acquired upon averaging 40 laser shots. The overall spectra were acquired at 5000–12,000 cm^–1^ with a spectral resolution of 64 cm^–1^. The emission spectra in Figure 1e,f were then obtained by integrating the time-resolved spectra within 1 μs after pulsed excitation.

### Front-Face Fluorescence Up-Conversion System

The ultrafast fluorescence dynamics were captured using a home-built fluorescence up-conversion system, as shown in Figure 2. A fiber laser (Kasmoro-1030-FR4, mRadian) provided a fundamental 40.3 MHz output at 1030 nm, which was split into two arms. The first arm was frequency-doubled to 515 nm via a β-barium borate nonlinear optical crystal (BBO, Type-I, 6 mm × 6 mm × 1 mm, θ = 23.5°, φ = 90°, EKSMA Optics) to serve as the excitation source, while the second arm maintained the 1030 nm fundamental as the gate beam after passing through a motorized delay stage (MODEL 2047, CDP System). To minimize the inner filter effect and self-absorption, a front-face collection geometry was employed. The 515 nm excitation beam at 260 mW was focused onto a rotating sample cell using an off-axis parabolic mirror (OAP, diameter = 2 in., focal length = 1 in., 45°, COHERENT), which simultaneously served as the collection optic for the resulting fluorescence. This back-reflected fluorescence passed through a long pass filter (RG-780, SCHOTT) to remove 515 nm laser scattering and other unwanted fluorescence and then focused onto a second BBO crystal (Type-I, 6 mm × 6 mm × 1 mm, θ = 29.2°, φ = 90°, EKSMA Optics) using a second OAP (diameter = 2 in., focal length = 2 in., 60°, COHERENT). Within this crystal, the fluorescence underwent sum-frequency generation (SFG) with the 1030 nm gate beam, which was focused by a D-cut lens (outer diameter = 1 in., focal length = 6 mm) to generate the fluorescence optical gating (FOG) signal. Notably, a D-cut lens was used to focus the 1030 nm gate beam to allow for a 12° crossing angle with the fluorescence within the limited spatial geometry. The up-converted FOG signal was guided through a pair of identical convex lenses (Outer diameter = 2 in., focal length = 10 mm) into a monochromator (MOD. 2022, CDP System) for wavelength selection. The FOG signal was detected by a photomultiplier tube (PMT, operating range: 190–900 nm, R955, Hamamatsu). To ensure that the FOG fluorescence signal had no other optical interference, a 560 nm band-pass filter (ZET561/10x, CHROMA) and a 532 nm long pass edge filter (LP03–532RE-25, Semrock) were placed before the detector to eliminate the second-harmonic generation (SHG) from the gate beam. Each spectrum was acquired using 80.6 million excitation pulses per time delay, and a signal-to-noise ratio (S/N) of 334 or higher was achieved. The instrument response function (IRF) was determined to be approximately 250 fs (full width at half-maximum, FWHM), evaluated by cross-correlating a 515 nm scattered light, via a diffuser (DGUV10–1500, Thorlabs), with the 1030 nm gate pulse (Figure 3).

**Figure 3:**
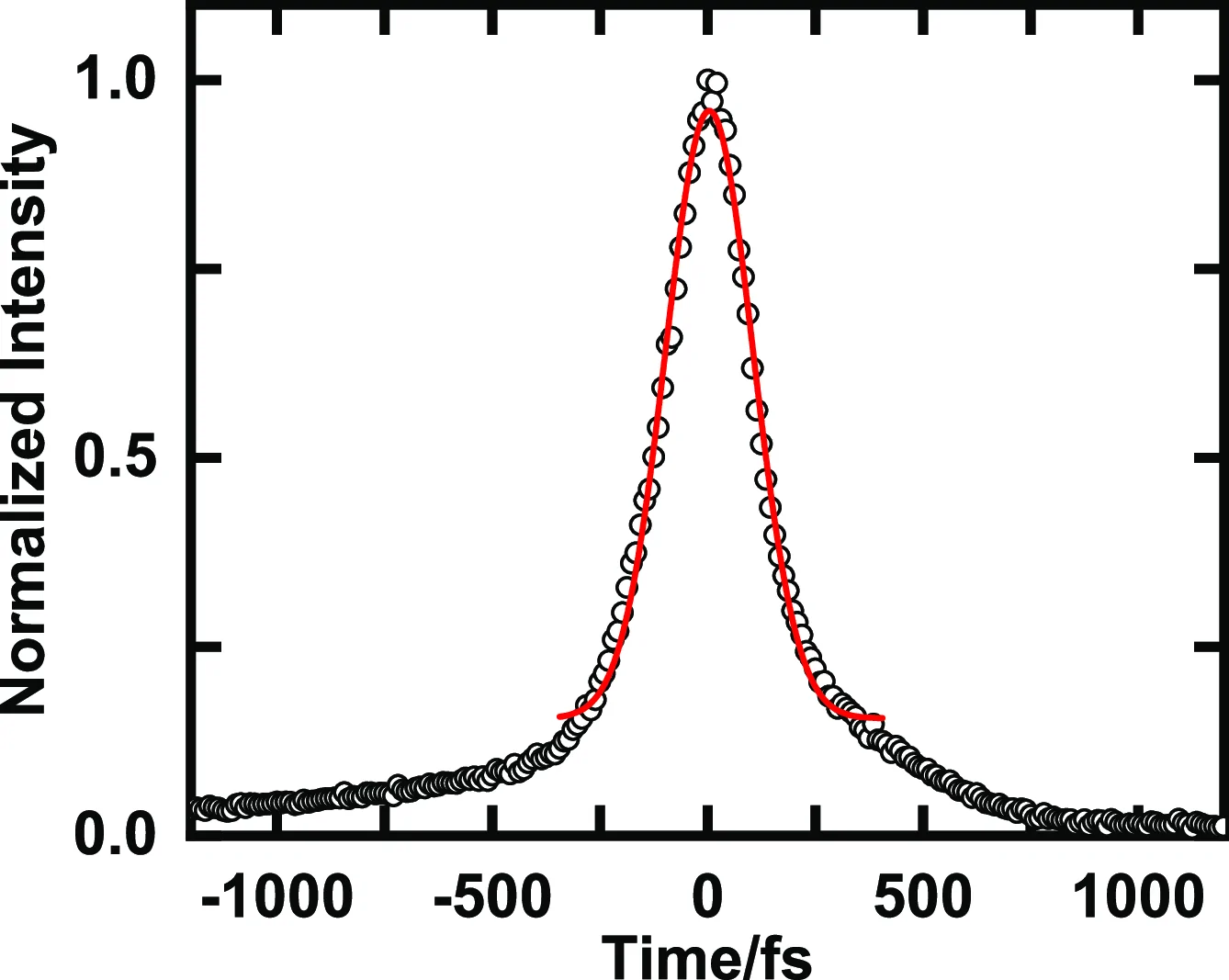
Normalized IRF profile. The system IRF measured by FOG is ∼250 fs (FWHM), determined by cross-correlating 515 nm scattered light with the 1030 nm gate pulse.

## Results and Discussion

The temporal profiles of the fluorescence up-conversion transients for the IR1061 and S2F-2H^+^ in DCM at 1235 nm with excitation at 515 nm are shown in Figure 4a,b, respectively. To maintain a consistent detection wavelength for direct comparison, IR1061 fluorescence was collected from the very weak shoulder band at 1235 nm (Figure 1e). Despite this, the S/N ratio of the IR1061 temporal profile remained sufficiently high (*ca*. 405), as shown in Figure 4a. The decay, fitted with a single exponential decay, was 31 ± 1 ps, in good agreement with the reported value of 39 ps by Prosposito et al.^26^ In the case of the S2F-2H^+^ fluorescence transient, the signal was monitored at the emission maximum of 1235 nm. As illustrated in Figure 1f, the absorption and emission spectra exhibited a significant spectral overlap with a minimal Stokes shift of approximately 64 cm^–1^. Despite the challenges in photon collection caused by the strong self-absorption inherent in its highly symmetric structure, the use of the front-face geometry FOG system allowed for the successful acquisition of the fluorescence profile with an exceptional S/N ratio of *ca*. 334. Although the traditional transmission geometry was also evaluated for S2F-2H^+^, it failed to capture any detectable signal due to heavy self-absorption, thereby necessitating the use of the front-face collection setup. The temporal profile, shown in Figure 4b, manifested a single exponential decay, in terms of a lifetime of 74 ± 3 ps, representing the relaxation from the S_1_ state to the ground state. The remarkably short fluorescence lifetime indicates that the relaxation dynamics are likely dominated by the nonradiative processes, in excellent agreement with the energy gap law, which governs the characteristically rapid nonradiative decay of near-infrared fluorescent dyes.^7,8^

**Figure 4:**
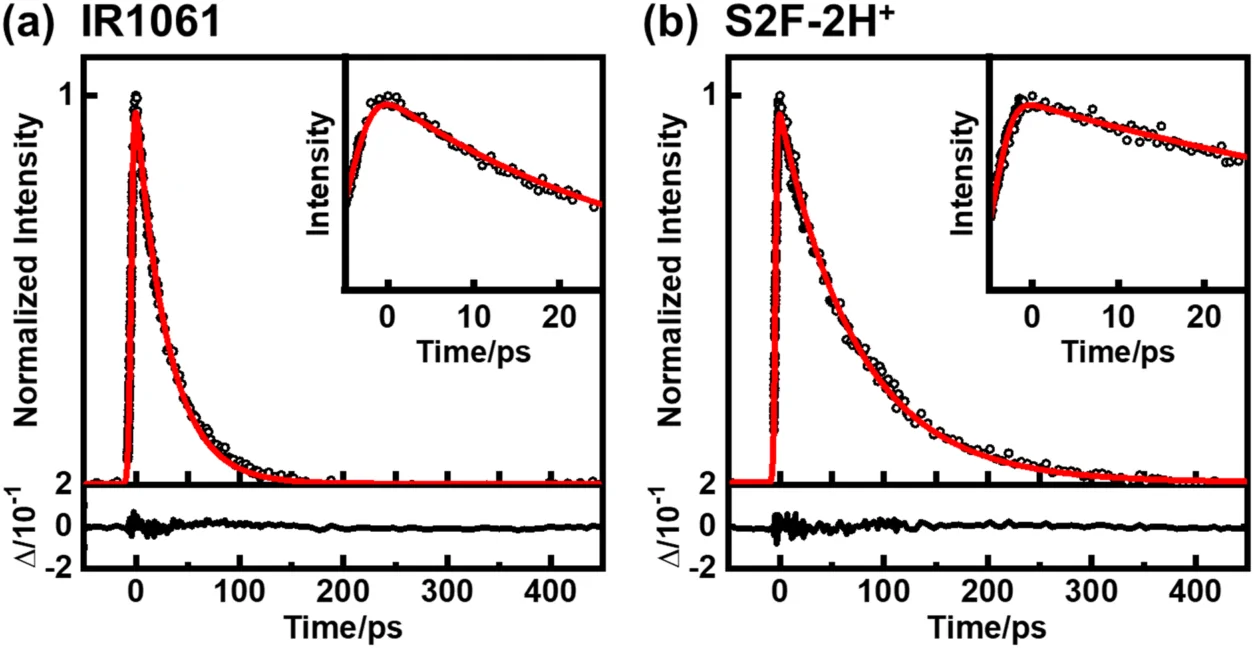
Normalized fluorescence temporal profiles of (a) IR1061 and (b) S2F-2H^+^ in DCM solutions at 1235 nm upon excitation of 6.5 nJ/pulse at 515 nm. Open circles and solid line denote the observed and fitting data, respectively. The fitted residuals, where Δ = observed–fitted, are shown in the lower frames. The inset shows an enlarged view in the −5–25 ps region.

While the system IRF at 515 nm is approximately 250 fs (Figure 3), shifting the detection wavelength to the near-infrared (NIR) region at 1235 nm causes the apparent IRF to significantly broaden to approximately 4–5 ps (Figure 4). The increase in the IRF at longer fluorescence wavelengths is consistent with the dispersive properties of nonlinear optical crystals, where the group velocity mismatch (GVM) between the interacting pulses leads to a significant temporal walk-off effect.^27,28^ This broadened IRF appears with both IR1061 and S2F-2H^+^, confirming that it represents an instrument-limited response rather than a manifestation of sample kinetics. Although an ultrafast S*_n_* to S_1_ internal conversion is expected for IR1061 and S2F-2H^+^, these processes typically occur on the subpicosecond time scale.^29^ Consequently, the intrinsic kinetic rise is entirely convolved within this 4–5 ps profile, which prevents the quantitative resolution of the true internal conversion dynamics. To improve the temporal resolution, employing a thinner nonlinear crystal will be essential to mitigate GVM-induced broadening in future measurements.^30^

## Conclusions and Implications in Analytical and Physical Chemistry

In this work, the implementation of the front-face optical configuration within the FOG framework effectively overcomes the challenge of inner filter effects in highly self-absorbing NIR-II materials.^13,14^ By circumventing the signal distortions inherent in traditional transmission geometries, this approach provides a robust and high-resolution platform for characterizing the intrinsic ultrafast dynamics of chromophores in the NIR-II range.

Time-resolved emission measurements in the NIR-II spectral range (900–1700 nm) have conventionally been performed using time-correlated single photon counting (TCSPC).^31^ While modern TCSPC systems utilizing advanced InGaAs single photon avalanche diodes (SPADs) have achieved continuous-mode operation and reduced dark count rates, their temporal performance remains fundamentally governed by the instrument response function (IRF), which is not better than 100 ps.^32^ This temporal resolution is primarily limited by the inherent response time and the relatively long dead time of infrared detectors, which often require up to 40 μs between detection events, as well as by the electronic jitter of the detection system.^31,32^ Consequently, while these methods are effective for characterizing picosecond fluorescence decays, the reported ∼70 ps lifetime of IR1061 via TCSPC after deconvolution may reflect instrumentation-limited resolution rather than the inherent decay time. As such, TCSPC encounters a “temporal bottleneck” when attempting to resolve faster, subpicosecond dynamics. In contrast, FOG measurements reveal a substantially shorter lifetime of 31 ± 1 ps for IR1061, which more accurately represents its intrinsic ultrafast decay.

On the other hand, step-scan Fourier-transform infrared spectroscopy (ssFTIR) enables the simultaneous acquisition of broad spectral and time-resolved data from a single measurement.^25^ ssFTIR has been widely used in unveiling reaction dynamics and photochemical reactions.^33^ Moreover, Shao et al. extended the approach to photoluminescence measurements of semiconductors, spanning spectral ranges from the NIR to the MIR.^34^ However, their NIR demonstrations were confined to the visible-NIR boundary (∼667 nm, GaInP/AlGaInP multiple quantum wells), leaving the NIR-II window (900–1700 nm) unexplored. Although the present work has demonstrated the successful applicability of ssFTIR on photoluminescence detection in the NIR-II window (900–1700 nm), the concomitant temporal resolution is fundamentally constrained by the inherent response times of infrared detectors, typically in tens of nanoseconds. Therefore, ssFTIR was used only for static (time-integrated) NIR-II emission, not for time-resolved measurements.

In contrast to the aforementioned methods, FOG spectroscopy offers significant performance advantages by leveraging SFG to convert NIR emission into the visible regime, effectively circumventing the electronic jitter and long response times of infrared detectors.^25,26,31,32^ Although broadband detection of the NIR emission examined here remains challenging due to the lack of current direct methods, FOG provides an efficient alternative. Specifically, the temporal resolution of FOG using a femtosecond gate pulse is no longer limited by the detector and electronics but instead determined by the laser pulse width and the delay stage length, offering subpicosecond resolution within a 1 ns temporal window. When the wide transparency of BBO crystals (up to 2 μm) is used, the NIR-II signal (900–1700 nm) is mixed with a 1030 nm gate pulse up-converted to the 491–577 nm range, which is sensitive to the multialkali-based photomultiplier for improved sensitivity and reduced thermal noise. Although FOG acquires a single wavelength per setup, its accessible range spans 900–1700 nm by tuning the BBO crystal angle, bandpass filters, and monochromator. This framework provides an efficient means of resolving early stage excited-state dynamics with high temporal resolution, which remain inaccessible to TCSPC or ssFTIR in the NIR.

## References

[1] Li C., Chen G., Zhang Y., Wu F., Wang Q. (2020). Advanced Fluorescence
Imaging Technology in the Near-Infrared-II Window for Biomedical Applications. J. Am. Chem. Soc..

[2] Antaris A. L., Chen H., Cheng K., Sun Y., Hong G., Qu C., Diao S., Deng Z., Hu X., Zhang B., Zhang X., Yaghi O. K., Alamparambil Z. R., Hong X., Cheng Z., Dai H. (2016). A Small-molecule Dye
for NIR-II Imaging. Nat. Mater..

[3] He S., Song J., Qu J., Cheng Z. (2018). Crucial Breakthrough
of Second Near-Infrared Biological Window Fluorophores: Design and
Synthesis toward Multimodal Imaging and Theranostics. Chem. Soc. Rev..

[4] Bin Y., Huang L., Qin J., Zhao S., Tian J., Zhang L. (2025). Exceptional Near-Infrared II Organic Small Molecule Nanoagent for
Photoacoustic/Photothermal Imaging-Guided Highly Efficient Therapy
in Cancer. Bioconjugate Chem..

[5] Zhao H., Li C., Shi X., Zhang J., Jia X., Hu Z., Gao Y., Tian J. (2024). Near-infrared II Fluorescence-guided Glioblastoma Surgery
Targeting Monocarboxylate Transporter 4 Combined with Photothermal
Therapy. eBioMedicine.

[6] Ren K., Li X., Zhu J., Yang H., Yang Z., Zhang F., You C., Xiang S., Tong Y., Chen J., Gao X., Kang M., He M., Li D., Fei P., Zhang Z., Wang D., Tang B. Z. (2026). Alkyl Chain Engineering
for High-Brightness Near-Infrared-II Aggregation-Induced Emission
Nanoprobes toward High-Resolution *in vivo* Bioimaging. ACS Nano.

[7] Englman R., Jortner J. (1970). The Energy Gap Law for Radiationless Transitions in
Large Molecules. Mol. Phys..

[8] Friedman H. C., Cosco E. D., Atallah T. L., Jia S., Sletten E. M., Caram J. R. (2021). Establishing Design Principles for
Emissive Organic
SWIR Chromophores from Energy Gap Laws. Chem.

[9] Ghosh P., Alvertis A. M., Chowdhury R., Murto P., Gillett A. J., Dong S., Sneyd A. J., Cho H.-H., Evans E. W., Monserrat B., Li F., Schnedermann C., Bronstein H., Friend R. H., Rao A. (2024). Decoupling
Excitons
from High-Frequency Vibrations in Organic Molecules. Nature.

[10] Zhao X., Zhang F., Lei Z. (2022). The Pursuit of Polymethine
Fluorophores
with NIR-II Emission and High Brightness for *in vivo* Applications. Chem. Sci..

[11] Liu D., He Z., Zhao Y., Yang Y., Shi W., Li X., Ma H. (2021). Xanthene-Based
NIR-II Dyes for *in vivo* Dynamic Imaging
of Blood Circulation. J. Am. Chem. Soc..

[12] Pan H.-M., Wu C.-C., Lin C.-Y., Hsu C.-S., Tsai Y.-C., Chowdhury P., Wang C.-H., Chang K.-H., Yang C.-H., Liu M.-H., Chen Y.-C., Su S.-P., Lee Y.-J., Chiang H. K., Chan Y.-H., Chou P.-T. (2023). Rational Design
of Asymmetric Polymethines to Attain NIR­(II) Bioimaging at > 1100
nm. J. Am. Chem. Soc..

[13] Sarma T., Kim G., Sen S., Cha W.-Y., Duan Z., Moore M. D., Lynch V. M., Zhang Z., Kim D., Sessler J. L. (2018). Proton-Coupled
Redox Switching in an Annulated π-Extended Core-Modified Octaphyrin. J. Am. Chem. Soc..

[14] Tsai Y.-C., Chen Y.-C., Lu H.-F., Chan K.-M., Lin S.-L., Lin P.-X., Rotomskis R., Steponkiene S., Wu T.-K., Chan M.-H. (2025). Energy Gap Law-Harnessing
Design of Highly Second Near-Infrared Emissive 34π-Annulated
Porphyrinoids for *in vivo* Imaging. J. Am. Chem. Soc..

[15] Lakowicz, J. R. Principles of Fluorescence Spectroscopy, 3rd ed.rd ed.; Springer, 2006.

[16] Maity N., Kim W., Panjwani N. A., Kundu A., Majumder K., Kasetty P., Mishra D., Bittl R., Nagesh J., Dasgupta J., Musser A. J., Patil S. (2022). Parallel Triplet Formation Pathways
in a Singlet Fission Material. Nat. Commun..

[17] Appavoo K., Sfeir M. Y. (2014). Enhanced Broadband
Ultrafast Detection of Ultraviolet
Emission Using Optical Kerr Gating. Rev. Sci.
Instrum..

[18] van
der Meulen P., Zhang H., Jonkman A. M., Glasbeek M. (1996). Subpicosecond
Solvation Relaxation of 4-(dicyanomethylene)-2-methyl-6-(p-(dimethylamino)
styryl)-4H-pyran in Polar Liquids. J. Phys.
Chem. A.

[19] Hoshi R., Suzuki K., Hasebe N., Yoshihara T., Tobita S. (2020). Absolute Quantum Yield Measurements
of Near-Infrared
Emission with Correction for Solvent Absorption. Anal. Chem..

[20] Zeng P.-R., Chu L.-K. (2025). Effective Species in UV/H_2_O_2_ Advanced
Oxidation Process: A Case of Degradation of 3,3′-Diethylthiadicarbocyanine
Iodide (DTDCI). ACS EST Water.

[21] Liu J.-L., Yang Y.-T., Lin C.-T., Yu Y.-J., Chen J.-K., Chu L.-K. (2017). Monitoring the Transient
Thermal Infrared Emission
of Gold Nanoparticles upon Photoexcitation with a Step-Scan Fourier-Transform
Spectrometer. J. Phys. Chem. C.

[22] Guo S.-S., Chu L.-K. (2018). Radiative Cooling
of Surface-Modified Gold Nanorods
upon Pulsed Infrared Photoexcitation. J. Phys.
Chem. Lett..

[23] Lu J.-Y., Chen H.-A., Yang C.-M., Chu L.-K. (2021). Radiative Relaxation
of Gold Nanorods Coated with Mesoporous Silica with Different Porosities
upon Nanosecond Photoexcitation Monitored by Time-Resolved Infrared
Emission Spectroscopy. ACS Appl. Mater. Interfaces.

[24] Lai J.-J., Shih M.-C., Chu L.-K. (2022). Influence
of the Thickness of Silica
Layer on the Radiative Relaxation of AuNR@SiO_2_ Core–shell
Nanostructures upon Photoexcitation. J. Chin.
Chem. Soc..

[25] Chu L.-K. (2023). Applications
of Time-Resolved Step-Scan Fourier-Transform Infrared Spectroscopy
in Revealing the Light-Initiated Reactions in Condensed Phases. J. Chin. Chem. Soc..

[26] Prosposito P., Casalboni M., De Matteis F., Glasbeek M., Quatela A., van Veldhoven E., Zhang H. (2001). Femtosecond Dynamics of IR Molecules
in Hybrid Materials. J. Lumin..

[27] Xu J., Knutson J. R. (2008). Ultrafast
Fluorescence Spectroscopy via Upconversion
Applications to Biophysics. Methods Enzymol..

[28] Chen Y., Cai Y., Xin X., Wang X., Huang K., Wu E. (2023). Low-noise
Synchronized Mid-infrared Upconversion Spectrometer with a Large Spectral
Coverage. Appl. Phys. Lett..

[29] Baskin J. S., Yu H.-Z., Zewail A. H. (2002). Ultrafast Dynamics of Porphyrins
in the Condensed Phase: I. Free Base Tetraphenylporphyrin. J. Phys. Chem. A.

[30] Rhee H., Joo T. (2005). Noncollinear Phase Matching in Fluorescence Upconversion. Opt. Lett..

[31] Becker, W. ; Shcheslavsky, V. In TCSPC FLIM in the Wavelength Range from 800 to 1700 nm; Proc. SPIE 9723, Reporters, Markers, Dyes, Nanoparticles, and Molecular Probes for Biomedical Applications VIII, SPIE, 2016 97230K 10.1117/12.2217500.

[32] Infrared InGaAs/InP SPAD Detector Information, Becker & Hickl GmbH 2026 https://www.Becker-hickl.com/products/infrared-ingaas-inp-spad-detector/. (accessed September 4).

[33] Chu, L.-K. ; Huang, Y.-H. ; Lee, Y.-P. Molecular and Laser Spectroscopy, Advances and Applications; Gupta, V. P. , Ed.; Elsevier, 2022; Chapter 14, Vol. 3, pp 481–527.

[34] Shao J., Lu W., Lü X., Yue F., Li Z., Guo S., Chu J. (2006). Modulated Photoluminescence Spectroscopy with a Step-scan Fourier
Transform Infrared Spectrometer. Rev. Sci. Instrum..

